# Factors influencing sustainable employment of persons with acquired brain injury (ABI) or spinal cord injury (SCI): A qualitative study evaluating the perspective of health and work professionals

**DOI:** 10.3389/fresc.2022.906567

**Published:** 2023-01-20

**Authors:** Monika E. Finger, Katarzyna Karcz, Barbara Schiffmann, Stefan Staubli, Margret Hund-Georgiadis, Reuben Escorpizo

**Affiliations:** ^1^ Work and Integration Group, Swiss Paraplegic Research, Nottwil, Switzerland; ^2^ Department of Health Sciences and Medicine, University of Lucerne, Lucerne, Switzerland; ^3^ Department of vocational Integration (ParaWork), Swiss Paraplegic Center, Nottwil, Switzerland; ^4^ REHAB Basel, Basel, Switzerland; ^5^ Department of Rehabilitation and Movement Science, University of Vermont, Burlington, VT, United States

**Keywords:** sustainable employment, spinal cord injury, acquired brain injury, work disability, social security

## Abstract

**Background:**

The number of persons with acquired brain injury (ABI) or spinal cord injury (SCI) who leave the labor market early despite successfully return to work post-injury, demonstrates the challenge for them to remain employed. Evidence on how enabling and hindering factors influence daily work across the lifespan and how they affect employment-related services is scarce. Professionals directly involved in work integration can add to this evidence through their experiential knowledge.

**Purpose:**

To identify and explore the factors that enable or hinder sustainable employment for persons with ABI or SCI from the perspective of health and work professionals.

**Methods:**

We conducted 23 semi-structured interviews with professionals in Switzerland, directly involved in work reintegration and retention of persons with ABI or SCI. Interviews were transcribed verbatim and thematically analyzed.

**Results:**

Participants identified three main themes related to the concept of “sustainable employment”. First, *the value and impact of initial work integration*; an early, multidisciplinary, person-centered work integration, with the early involvement of employers is ideal. A good match between the worker and the workplace is sought. Second, *critical factors for long-term sustainable work*: the main risks for persons with ABI are changing supervisors, workplace restructuring and the introduction of new technologies, while deteriorating health and the occurrence of secondary health problems are the greatest risk for persons with SCI. Third, *the relevance of knowledge, experience and attitudes of professionals*; Knowledge of the consequences of an ABI or SCI, the legal basis and the social security process, and the attitude of professionals towards the injured worker were considered important.

**Conclusions:**

From the professional's perspective, enabling and hindering factors for sustainable employment in the long-term are fundamentally very similar for persons with ABI and SCI. But different physical, mental and neuropsychological effects call for individually adapted measures. While persons with SCI primarily require ongoing medical care, conscious management of changes in the workplace is critical for persons with ABI. For both groups, an easily accessible counseling and support service should be established for work-threatening problems in the long-term. Furthermore, diagnosis-specific training programs for professionals of employment-related services and disability management should be developed.

## Introduction

Being engaged in gainful work provides economic security and the workplace being an important place of social integration for the majority of people in our society (1). This is also true for persons with a disability (2). The United Nations considered the “right to work” of persons with a disability when adopting *the Convention on the rights of persons with disabilities (CRPD)* in 2006 in Article 27 “Work and Employment”. This article begins with, “States parties recognize the right of persons with disabilities to work on an equal basis with others.” Although by 2022, 185 states of 193 countries globally have ratified the CRPD, the labor reality of persons with a disability is still challenging, and it can be very difficult for them to find and (perhaps more importantly) maintain a job, even in high income countries such as Switzerland (3).

Spinal cord injury (SCI) and acquired brain injury (ABI) are prototypical examples of neurological disorders and are among those injuries that cause a significant amount of years of healthy life lost due to disability (YLD) worldwide (4). SCI affects the spinal cord and ABI affects the brain – and while they affect different structures of the nervous system, together they cover the vast majority of movement-related, neuropsychological, psychological, and social functioning-related problems, typically associated with neurological disorders (5–11). Common aspects that may become apparent can possibly be generalized to other types of disability. Differences refer to particularities of the respective group and its environment. These differing factors should be analyzed for the group in question and individual measures derived based on the results.

The WHO Rehabilitation Needs Estimator estimates that there are approximately 73 million people of working age living with an ABI and 14 million with an SCI worldwide (12, 13). The employment rates of persons with ABI range between 35% and 71% depending on the study population, country specific regulations and definitions of work. The employment rates of persons with SCI range from 10.3% to 61.4% (14). Focusing on Switzerland, there are approximately 90'000 persons in working age living with an ABI, which corresponds to an age dependent prevalence of 490–1792 per 100,000 persons for traumatic brain injury and 140- 1,300 for stroke (13, 15). Data on employment rates of persons with ABI are missing. Roughly 6,000 persons with an SCI live in Switzerland, which 62.8% of whom are of working age. For SCI, an employment rate of 61.4% was found based on a national cohort study (SwiSCI) (16). In an international comparison, this employment rate is one of the highest, but it is still well below the employment rate of the general population which is 78.6% (17).

In recent years, great efforts have been made in Switzerland, through legislative revisions, to integrate persons with SCI or ABI after their injury back into the labor market (18).

Thanks to growing evidence of factors that can predict labor market participation and factors that promote vocational (re)integration (19–23), new services in vocational rehabilitation have been created. In Switzerland, rehabilitation for people with SCI, including long-term inpatient and outpatient care, is provided primarily in four specialized SCI centers and follows an integrated care model. ABI rehabilitation is more fragmented and is offered regionally in several acute hospitals, inpatient and outpatient rehabilitation clinics and practices. These services, with a focus on early vocational intervention, workplace adaptations and aids for work, are financed by the national disability insurance (DI). The DI also pays disability benefits. The amount of these benefits is based on the disability-related level of loss of earnings and not exclusively on the medical diagnosis and the resulting impairment.

Despite these major integration effort, long-term data from the United States show that a significant share of persons with SCI or ABI who have successfully returned to the labor market, end up leaving work before reaching retirement age (Retirement age in Switzerland is 64 for women and 65 for men) (24, 25). Evidence of early exit from work comes from the two patient organizations – the SCI-centric Swiss Paraplegic Association and the ABI-centric FRAGILE Suisse -, which both report an increasing number of requests for help from persons with ABI or SCI who contact them after losing their employment (19, 20).

Because quality evidence that is based on a life course perspective, as well as evidence that focuses on factors critical for gainful employment for persons with ABI or SCI throughout their work life is still scarce, three of the authors (KK, MF, RE) conducted two diagnosis-specific scoping reviews. These reviews identified factors that can help to understand why some persons succeed at work after their injury and why others have poorer outcomes (21, 22), and factors associated with long-term employment of persons with ABI or SCI from an international perspective. However, they also showed a lack of inept information on how these factors directly impact the working life of the concerned population and how these factors might contribute to a sustainable work situation or what kind of support would be needed over time to maintain work activity. A sustainable work situation can be defined as: “a person–job–workplace match that enables a person to stay healthy and satisfied at work over time, with a work performance that meets the expectations of the person and the employer” (23). Results of the reviews also point to country specific influences, as shown by the different employment rates of persons with a disability (14). Since there are few qualitative studies for Switzerland assessing the work reality of persons with SCI (26) and none for persons with ABI, we conducted two parallel studies asking persons with ABI or SCI and employers of people with ABI or SCI about facilitating and hindering factors for sustainable work.

Affected workers emphasized the impact of impairment-related limitations on their work performance, such as limited mobility and pain problems in SCI and problems with excessive fatigue and limited cognitive resources in ABI, as well as the importance of an adapted workplace and work organization. They also rated supportive private and work environments as critical, followed by personal skills in self-advocacy, communication, and learning how to live with a disability. Pending insurance decisions and problems with social security, on the other hand, were perceived as barriers (27). Employers identified four main issues: sociodemographic and psychological characteristics of the disabled person, his or her work performance, the work environment, and other social/environmental conditions. They considered good self-management of the disability and proactive communication of needs on the part of the employee to be important. Differing expectations and assessments of job performance by employees and employers, on the other hand, pose a challenge. Employers feel a responsibility to provide an optimal work environment that allows the employee with a disability to reach his or her full potential. Both, employees and employers, reported that the support of professionals during the work integration phase was important for achieving sustainable employment, as they often do not have the knowledge to design the work environment to meet the person's needs.

In addition to the experiences of persons with ABI or SCI and their employers, professionals directly involved in vocational integration or maintenance of persons with a disability can provide a comprehensive view of current services, service gaps, interactions among professionals, and an external perspective on the employee-employer relationship.

### Research aim

The aim of this study was to identify and explore the factors that enable or hinder sustainable employment for persons with ABI or SCI in Switzerland from the perspective of vocational integration, long-term care, and insurance professionals.

Our specific aims were (i) to explore factors that promote or hinder persons with SCI or ABI to stay sustainably at work after their injury, and (ii) to identify persons or services that may provide guidance or assistance for the injured workers if necessary during their work life.

## Methods

We applied a qualitative design using semi-structured single interviews. Data was thematically analyzed using qualitative content analysis (28).

### Recruitment strategy and sample

Participants were invited to represent a comprehensive sample of professionals with insight into the work-life situation of persons with SCI or ABI. They are expected to know the critical influencing factors for sustainable employment, and have insight into how these factors may influence each other. In addition, professionals are expected to be familiar with the existing (and possibly missing) support services, the administrative processes, as well as the legal basis of the social system.

Inclusion criteria were: trained professionals with experience in the vocational reintegration of persons with ABI or SCI, or both, and with insight into their further work-life aspects, ideally over the entire work-life span until retirement. The sample included health professionals, work professionals and vocational rehabilitation specialists, advocates, professionals from advisory agencies, representatives of patient organizations and persons representing private insurance companies or the Swiss national disability insurance (DI) to ensure diverse perspectives. To promote data saturation, we recruited at least two persons from each stakeholder group, with equal representation from SCI and ABI. Data saturation is reached when no new information is obtained during the interviews (29).

We used several recruitment strategies including:

• recruiting professionals by flyers posted in an online forum for persons with SCI and their families and health professionals, (www.community.paraplegie.ch), and the web page of Fragile Suisse, the organization for persons with brain injuries, their relatives and professionals (www.fragile.ch).
• recommendation from two vocational rehabilitation departments, the work integration interest group (BRIG) of the Swiss association of rehabilitation (SAR);
• and through personal contacts of the first and second author.

Potential participants were sent a study flyer requesting them to contact the study team if they are interested to participate. If interested, one of the authors (MF, BS) verified the inclusion criteria and provided study information by phone. All professionals, who meet the inclusion criteria agreed to participate. ATan appointment was made for the interview and written study documentation was sent by e-mail. Potential participants were also asked for further recommendations and to distribute the study flyer to their network.

### Data collection and analysis

MF (a trained physiotherapist experienced in qualitative research, PhD) and BS (a sociologist experienced in qualitative research, MSc) collected data by expert interviews based on a semi-structured interview guide (Supplementary material 1). The guide begins with the interviewers introducing themselves with their professional background, their role in the project, and their personal interest in the topic. The interview then started with generic open questions on the following topics: information about the participants’ background; his or her experience with persons with ABI or SCI, and factors and barriers that influence sustainable employment; commenting on main themes from a literature review on facilitators and barriers for employment after SCI or ABI (personal characteristics, work performance, work environment and general environment) that were presented by the interviewer (21, 22). Finally, the professionals were asked where they thought a person with increasing problems at work or problems with his or her employer could find support after the work integration period has ended.

In total, twenty-three professionals participated in this study; 10 with a focus on working with persons with ABI or with SCI and three professionals with experience with both conditions (Table 1). Most interviews were conducted at the workplace of the professional, two interviews took place at the research institute (SPF) and three interviews were conducted over Zoom.

**Table 1:** Participants characteristics.

Participant number	Diagnostic Focus	Years of work experience	Main Phase	Profession and work experience
1	ABI	10	Integration	Vocational specialist In- and outpatient rehabilitation
2	ABI	8	Integration	Vocational specialist Outpatient rehabilitation
3	ABI	9	Integration and long-term	Consultant Patient organization, consulting
4	ABI	10	Integration	Job coach, NGO
5	ABI	>5	Integration and long-term	Patient advocate
6	ABI	34	Integration and long-term	Physician, neurologist
7	ABI	24	Integration and long-term	Social Counselor, NGO
8	ABI	8	Integration	Job coach, self-employed
9	ABI	15	Integration	Job coach, self-employed
10	ABI	10	Integration	Vocational specialist In- and outpatient rehabilitation
11	ABI & SCI	8	Integration	Case manager, disability insurance
13	ABI & SCI	14	Integration and long-term	Administrator Blended counselling, disability insurance
14	ABI & SCI	16	Integration and long-term	Vocational specialist, In- and outpatient rehabilitation
15	SCI/ABI	12	Integration and long-term	Case manager, accident insurance
16	SCI	15	Integration and long-term	Case manager, private insurance
17	SCI	32	Integration and long-term	Vocational-specialist, consultant
18	SCI	30	Integration	Work-psychologist, Inpatient rehabilitation
19	SCI	>5	Integration	Vocational counselor, In- and outpatient rehabilitation
20	SCI	16	Integration and long-term	Physician, Outpatient clinic
21	SCI	8	Integration and long-term	Physician, paraplegiologist, Inpatient rehabilitation
22	SCI	12	Long-term	Family physician
23	SCI	12	Integration	Case manager, accident insurance

Abbreviation: ABI, acquired brain injury; SCI, spinal cord injury.

Interviews ranged from approximately 35 to 100 min with an average of 50 min.

Interviews were audiotaped, transcribed verbatim and then analyzed using thematic analysis as described by Schreier (30). In addition the interviewer took notes on the course of the conversation and on relevant topics. First, transcripts were read several times by the first and third author (MF and BS) to get familiar with the data as basis to build a coding frame. The coding frame combined concept driven and data driven approaches (31), meaning that the construction started with the four ICF-component associated dimensions “injury related factors”, “functioning and skills”, “personal factors” and “environment”. The frame was then refined with a time code indicating if the identified factors related exclusively to the time of vocational rehabilitation and work integration after the injury, or if the factors related to the time of sustained employment several years after the end of reintegration. Within this general frame, MF coded the interviews and formed categories inductively as they emerged from the data. The data under each category was rephrased and summarized. MF went back frequently from coded text to the original transcript or sometimes even the audiotape to ensure that the categories reflected the original information. The meaning and interrelationships of the categories were discussed, and based on this, themes and subthemes were formulated in the research team (MF, KK, BS). Quotes were used to illustrate the themes. To code all interviews, MaxQDA 2020 (32) was used.

Finally to enhance trustworthiness, we presented the results of the interviews in three meetings to health professionals, work and integration specialists and professionals from the patient organizations. Participants included interviewees and non-participants.

To increase trustworthiness, we presented the interview results in three sessions to health care professionals, work- and integration professionals, and professionals from the two patient organizations. Participants included interviewees and individuals who did not participate in the study.

Ethical approval was obtained from the ethical committee of North-West and Central Switzerland (EKNZ, study reference 2018-01317). All participants gave written informed consent. Data was anonymized for privacy and confidentiality.

## Results

The analysis of the data revealed that depending on their daily work experience, participants – independent of their professional diagnostic focus - when referring to “sustainable employment”, talked about two different time points. First, professionals mainly working in the phase of VR or work integration (WI) after the injury, used the term “sustainable employment” interchangeably with “a stable work situation at the end of the integration process”. They understood sustainable employment as a situation in which all open questions concerning work organization, the relationship with employer and colleagues, as well as questions related to insurance and benefit issues have all been sorted out, and in which the work performance of the worker matches the expectations of the employer. They indicated that reaching a sustainable work situation is the ideal moment to end their professional engagement. Second, professionals with more overview of the whole working life of persons with a disability, understood “sustained employment” as a stable work situation over the years, with work as a natural part of the person's life. Nevertheless, working in the long-term – *several years after the end of first work integration*-, became only a topic for any of the professionals in their work, if a stable work situation is interrupted, a job loss became imminent, or the persons already lost their employment.

**Figure 1:**
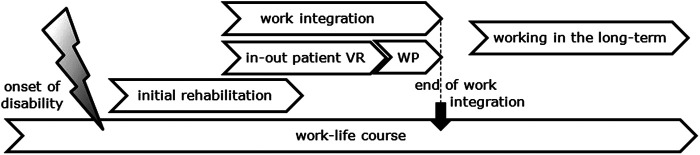
Process of working with a disability in Switzerland (VR, vocational rehabilitation; WP, work participation).

According to the two different periods of working with a disability addressed by the professionals – work integration and work life in the long-term -, we generated two major themes. A third theme that affects both phases equally addresses “the knowledge, the experience and the attitude of the professionals” (Figure 1).

All three themes and their subthemes were identified by the professionals for both ABI and SCI as significant and described with similar main arguments (Table 2). However, the relevance of individual subthemes was sometimes weighted differently for ABI and SCI (Supplementary material 2).

**Table 2:** Main themes and subthemes.

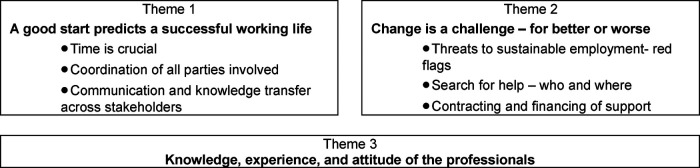

### Theme 1: A good start predicts a successful working life

Participants expressed the importance of a coordinated vocational rehabilitation and work integration phase after SCI or ABI as basis for a sustainable working life in the long-term. Participants from all professional groups agreed on the ultimate goal, which is to support the injured worker to return to a sustainable gainful employment. Vocational rehabilitation and work integration specialists focused strongly on a patient-centered, individualized process and Supported Employment strategies (33), as described by a job counselor:

*N.: 14: Vocational specialist*

*For me, ideal professional coaching as part of professional reintegration looks like this: There are four to five phases. First there is an order clarification phase, by which I mean that the prerequisite for me to become active is that the client wants to return to work and wants my support. (…). Already in this first step, the payer (private insurance or DA) is involved in the clarification of the order. The clarification phase is followed by profiling: What skills and resources does this person have? (…) Then, in the acquisition phase, we look specifically for a potential employer, an internship provider or a trial internship, where we then coach the patient in the work trial, in the internship. At the workplace, we provide integration support to secure the job and ensure that a permanent position results from the measure. This also includes helping the client with the issue of benefit assessment or case closure pension review.*

*The process I have just described is nothing other than the Supported Employment model*.

Insurance and social system representatives were additionally interested in a general overview of the work integration process and focused also on the associated financial, administrative, and legal procedures. This overview usually starts with the first vocational measures in the initial rehabilitation phase and ends with the completion of the work integration phase, in which a final work capacity is officially determined and any disability pension is decided.

*Nr.13 Administrator disability -insurance*

*I think it's important to use all the possibilities [for early vocational integration] from the disability insurance side; with preparatory measures, work trials, vocational clarifications and so on. And that the measures can also be taken over a certain period … certain problems, I think, only arise after a certain period*.

#### Time is crucial

One of the major aspects in theme 1, illuminated from the responses of several participants was the *onset* and the *duration* of vocational integration.

Several professionals pointed out that the topic of *work activity* should be addressed as early as possible, ideally during their first medical rehabilitation session. These professionals made this recommendation for two reasons: First, they argued that an ABI or SCI is a major life-changing event, that requires a great effort on the part of both, the person and those around them to learn how to cope with this new life situation. In rehabilitation, persons with ABI or SCI often focus on getting well again and mastering the activities of daily living. When professionals ask these injured workers questions about their future employment and offer guidance on the process of vocational integration, it can help these persons come to terms with their life plans and find the courage to set participation goals outside of the rehabilitation center. Professionals also pointed out that psychological and personal characteristics, such as a strong motivation to work, personal life goals, and an ability to self-reflect and self-advocate play a strong role in the success of the reintegration – topics that may be addressed during their first rehabilitation period.

Although most vocational intervention-professionals advocated for an early contact with the injured person, they also emphasized the importance of giving the person the time to be physically and mentally ready to deal with the future and with professional aspects before returning to work or starting a vocational integration program, as illustrated in the following statement.

*Nr.18 Work-Psychologist*

*My main activity is to support persons in coping with paraplegia, which in turn also has to do with the ability to work. Because if someone cannot accept his disability, then he will have a hard time finding a job. Because in this case, his top priority will be to get healthy and not to work. Persons say this explicitly: My priority is to get healthy, everything else doesn't interest me*.

However, although professionals strongly supported such an all-encompassing vocational integration approach as ideal, they also reported that the nature and accessibility of vocational rehabilitation interventions in reality depends heavily on whether vocational interventions were organized as an integral continuing component of an initial rehabilitation program or not. If vocational interventions are not considered until the patient is discharged from the rehabilitation center or acute hospital, or if vocational rehabilitation is considered only when problems arise in returning to the old job, this makes successful sustainable work integration much more difficult. Delayed vocational interventions were reported mainly by persons with ABI discharged home directly after acute hospitalization:

*Nr.16 Case Manager, private insurance*

*Unfortunately, what I've seen with brain injury patients in recent years is that persons are discharged home [from the acute hospital] without checking with specialists to see if they're cognitively okay. And we [case managers from insurance] have to find out later, … , that there's something wrong with them. That's when vocational integration becomes very difficult*.

In addition to evaluating the perspective of the injured person, work professionals emphasized the importance of contacting employers early in the process, with the aim to retain former employment and to engage employers and colleagues in the rehabilitation process, as illustrated by the following quotes:

*Nr.2 Vocational specialist*

*It's very important for me to contact the employer early on. Simply to make it clear: “We are here, we are an institution, we accompany the return-to-work”. Because employers, like patients, are laypersons with the new situation, and that's why I think it's very important that information, and regular exchange can take place*.

*Nr.8 Job coach*

*The first time after the event is important and has a longer-term effect. 5–10 years later, many workers still like to think back: “There in the rehab, the boss and the whole team visited me. They made me a banner, came into the room, and thought of me”. These are long-term values that are not without significance and you never forget things like that*.

Information early on, on the expected timeframe for the worker to return to work and on the course of the integration process, enables the employer to organize its business operations accordingly. Realistic performance expectations on return during the on-the-job integration process also reduce the pressure that employers may put on workers. Professionals also reported that employers who agreed to support an injured worker on his or her way back to work, were often very supportive in the long-term. This was especially true for small and medium-sized companies, where workers have close personal relationships with the employer:

*Nr.21 Physician*

*I remember many employers very positively. Many of our patients' employers are very understanding. They look closely to see if there is a possibility that the former employee can return to their company. I find that very, very good*.

*Nr.2 Vocational specialist*

*It must be clear [for the employer] from the start that we are not talking about two or three weeks, but we are still in the rehabilitation phase. It is also about giving a brief outline of how such a return-to-work process proceeds, […] The patient starts slowly with reduced work load, but there are no costs for the employer in the first moment, as the insurance pays the worker's income. […] only when the performance is really there, then the work incapacity is adjusted*.

#### Duration of interventions

The second time-related aspect, *duration of interventions*, is associated with the time needed to reach a sustainable work situation. This topic was raised mainly by work integration specialists from both diagnostic groups and included several related topics. The duration of integration is caught between the legal framework regarding the financing of integration measures, the individual needs to recover and rebuild work capacity of the person concerned, and the requirements and options set by the employer.

Vocational integration measures are mainly financed by the Swiss disability insurance (DI). However, in most cases of early intervention, other payers such as accident or liability insurance or health insurance companies are also involved (34). Different financial responsibilities and interests of insurers as well as different practices, depending on the canton or even the insurance agency, can lead to unclear legal situations and delays or rejections of cost coverage. In summary, the financing of work-integration measures depends on a proper application to the insurance company, the insurance coverage of the worker during initial rehabilitation (medical and inpatient vocational rehabilitation after the injury), and the assessment of future earning capacity by the DI after discharge from initial rehabilitation. Time to a decision is mostly due to administrative procedures, as reported in the following quotes from a patient lawyer, and a case manager from a private accident-insurance:

*Nr.5 Patient advocate*

*With social security, it is sometimes the case that very complicated procedures must be followed, which also take a very long time. And I think that time is an important factor in successful reintegration*.

*Nr.15 Case manager, accident insurance*

*With persons with SCI, it is more that the infrastructure must be there so that one can start. And there too - the infrastructure is paid for by the DI and the employer sometimes pays for it in advance and then doesn't know whether they will get everything back. That's a bit of a financial risk for the employer. In some cases, you have to rely on goodwill so that the timing fits, because otherwise you could wait for years*.

Vocational specialists and case managers expressed the importance of having enough time to sustainably integrate persons with ABI or SCI. While professionals emphasized the individual length of time needed for persons with ABI to adapt to increasing workloads, such as working hours, the complexity of work tasks in their former profession, and if the work tasks are possible at the former workplace, SCI specialists often referred to time needed to retrain for a new profession:

*Nr.10 Vocational specialist*

*For subsequent long-term employment, it is important to take the time for integration. It's not usually done in three months. It really takes time; you have to look closely. A brain injury is not like a broken arm that usually grows back together right away. (…). But that is the advantage of today's DI, with the mandate of integration before disability-pension. That gives us a bit more time*.

In the context of work integration, professionals also emphasized the value of a long-term, easily accessible offer of support or a defined contact person for the worker and the employer to reflect on the actual situation, to increase workload in a controlled manner, or to consult in case of problems. Work counselors assisting persons with ABI also mentioned the challenge of being reimbursed for integration support over an extended period of time, as described in the following quote:

*Nr.8 Job coach*

*The social security system is exhausted at some point, and according to certain laws, certain measures are limited in time. Then it's over with professional support. Follow-up support for the employer, for example, or rather for the work context, i.e. for the worker and the employer, would often be extremely helpful*.

Financing long-term support was not discussed by professionals working with persons with SCI.

Experts called for long-term work integration support mainly to rebuild the worker's best possible work capacity in manageable steps. This re-building included the ability of the person to feel their limit and to know what they need to recover timely. They also had to establish a sensible work life balance by taking into consideration their physical, neuropsychological, and mental problems and limitations. Professionals working with persons with SCI consistently cited physical retraining, re-learning self-care and skin care, and vocational reorientation as a main reason for a long integration time. The main issue for persons with brain injuries was their need to build up their cognitive and physical endurance and work capacity in a controlled progressive manner, as shown by the following citations:

*Nr.19 Vocational counselor*

*The biggest challenges for work-integration? Hm. Yes, I think this is the pace of the integration process. I think there always has to be a pace that the patient can do. A pace which is theirs. After all, it's their path*.

#### Coordination of all parties involved

Repeatedly, participants outlined that successful work integration was closely related to a well-coordinated, interdisciplinary approach. They addressed mainly two aspects of interdisciplinary coordination: coordination across settings along the vocational integration process and coordination among stakeholders within a reintegration process or program.

Professionals from early integration as well as professionals from the insurance agencies and from patient organizations indicated that a coordinated vocational integration should ideally start during or directly after discharge from the first rehabilitation:

*Nr.9 Job coach*

*It is true that after medical rehabilitation, the DI starts with vocational rehabilitation. The cooperation with the specialized SCI clinic (SPZ) is extremely helpful here, precisely because of the well-coordinated interdisciplinary aspect*.

Although such a procedure was reported to be standard for some rehabilitation centers, professionals also reported that especially for persons with ABI with or without minor physical impairment, discharge from acute hospital without a defined return-to-work procedure is not uncommon.

*Nr.13 Administrator, disability insurance*

*It's just the question in the rehabilitation clinics how the case management is organized. But I think that a follow-up solution [addressing work integration] can be found there. It would be helpful if persons don't have to orient themselves from home alone: Who now? Where do I have to go? It is simply important to plan the transitions*.

The second aspect regarding coordination of vocational integration concerned the lead and the responsibilities within the stakeholder group. Depending on the setting, three major scenarios were discussed. *First, in the most preferred scenario was that vocational integration processes were directly initiated from work integration specialists during or after first rehabilitation as ongoing measures.* This was mainly the case, when rehabilitation clinics had a mutual agreement with an accident insurance or the DI with a formalized procedure on how and when to involve representatives from the insurance to get a formal contract and financial agreement to fund the work integration process. Scenario one affected almost exclusively persons with SCI. *The second scenario was that at the end of the acute medical treatment or first rehabilitation, further occupational measures were recommended in the discharge report to the payers (accident insurance, health insurance), and the family doctor.* It was then up to the administrative responsible-person of the payers, and the doctor together with the person concerned, to inform to apply for return-to-work measures. Such measures usually started with an assessment of the work-situation by a DI-case manager, as basis for further steps, such as career counseling, DI-case management, or to mandate an external case manager. This scenario involved both, persons with ABI and with SCI. *In the third case, in absence of VR, the workers themselves or the employers searched for support because of problems at the workplace after return-to-work, either from a patient organization, a health professional (family physician, neurologist or a therapist) or the DI directly. This scenario was almost exclusively mentioned in connection with persons with ABI*.

In all cases, the involved professionals emphasized the importance of an overall understanding of the situation and a final agreement on the roles of the involved stakeholders and the measures taken towards work integration, despite various perspectives, as illustrated by the following quote:

*Nr.23 Case manager, accident insurance*

*… it is also very important that the professionals around the injured worker, such as the responsible person from the accident insurance, the DI or the job coach, have regular contact and inform each other, and not that everyone tends his own garden*.

Although a coordinated return to work process including all stakeholders involved was emphasized by most professionals, there were also persons that criticized the increasing amount of time they had to invest and that other professionals were undermining their competencies, as expressed by a family doctor:

*Nr.22 Physician*

*The real problem is that we, doctors no longer have any status at all. A health insurance company has the right to refuse a sick note. I can write a note, and then some administrator says, No, that doesn't apply. My medical decision is just more and more questioned*.

*And there is this story of the case managers, who make sense from an ideal point of view, but are just a nuisance for me. They take over [the case] and work with their own systems, and I end up with even more work than I already had. If we then also have to talk to them, and if they then even organize some meetings and expect us to attend, then there is additional burden. So, my job is becoming more and more difficult because more and more bureaucracy and structures are being put in place. And that affects both the person who uses a wheelchair and the person who is able to walk*.

#### Communication and knowledge transfer across stakeholders

One major goal of the coordination of stakeholders involved is the direct exchange of information and further, based on a comprehensive common understanding of the situation, to agree on a coordinated vocational integration strategy.

The other goal was aimed at knowledge transfer or education and was considered extremely important, especially by job coaches, career counselors, and case managers. Several experts stressed the need to involve not only the worker and the employer, but also the person's co-workers in the reintegration process, to create an understanding of the needs and strength of the worker, as one vocational specialist stressed:

*Nr.2 Vocational specialist*

*Employers and co-workers, like patients, are lay persons with the new situation and therefore I find it very important that good Information, and a regular exchange can take place*.

To raise awareness and understanding, the job coaches addressed the topic of a brain injury or an SCI in general as well as the problems of the involved worker in particular - provided that the involved worker had given prior permission. Especially in the case of *invisible impairments*, knowledge of the consequences of ABI or SCI has proven to be a strong basis for co-workers to permanently accept the adapted working conditions of their colleagues with a disability, such as short-time work, flexible working hours, and reduced workload. *Invisible impairments* mentioned for SCI addressed mainly the management of continence, skin relief, and in case of an incomplete injury, gait and balance problems and the additional time needed for daily activities and exercises. The professionals working with persons with ABI emphasized the influence of cognitive impairments such as reduced attention, concentration and self-reflection, speech problems, fatigue and a longer recovery time, and non-supportive work environment as major threats to work performance.

*Nr.14, Vocational specialist*

*I have to support the work environment, the work colleagues, the supervisor, and the company to understand, how to deal with a person in a wheelchair. I have to develop an understanding that persons in wheelchairs not only use wheelchairs, but very often have a problem with defecation and urination and that it can also happen that someone has to leave work because of a pressure ulcer*.

*I don't want to scare persons, I want to educate them and to point out: “Hey, this is a guy, you can expect performance from him, but there are some specifics.”*

The job coaches also emphasized that they first took the perspective of the worker and often acted as a mediator between the person and others to create understanding and support and to mediate in case of conflict. However, they also mentioned the importance to understand the needs and concerns of the employer and to take these concerns seriously in order to then work together on individual solutions.

*Nr.4 Job coach*

*So I wear different hats sometimes. I am not a job coach from the DI, with the focus: “Somehow it must work”, but my first focus is the person concerned. It can also be that I do more specialized coaching for the management or the whole team, that my coaching then becomes more of a training. I offer, if my client is able to, that we do further trainings for the team together. This approach creates more understanding, and we can see which topics are burning and how we can address them*.

### Theme 2: change is a challenge

When asked about factors that enable or hinder sustained long-term employment after the end of the integration period, participants demonstrate little insight into the long-term work reality of persons with ABI or SCI. The professionals mainly mentioned that they were contacted primarily when there was a deterioration in health or major changes in the workplace by the affected employee or employer, when they realize that these problems cannot be solved without the help of a professional in the workplace. Changes *for the better* relate to career development and moving to a more suitable job - but these were only addressed for persons with SCI, while persons with ABI often struggle to keep up with evolving workplace demands. As reported by professionals, it is difficult for people with a disability to meet the requirements of lifelong learning, because vocational training and courses often last too many hours at a time, provide too much information at once, and in official training courses there is no possibility to adapt the content of the training or the time frame to the learning needs of the person concerned.

#### Threats to sustainable employment- and red flags

Professionals reported critical health changes impacting the work capacity of persons with SCI as related to a decrease in physical abilities, problems with infections, or an increase in neurologic and musculoskeletal pain, with the main “red flags” of increasing sick days, or having to go to a medical appointment. Participating physicians reported that if they identify health or environmental changes that have a long-term, negative impact on the patient's work situation during the annual health checks in the specialized SCI center, they are required to offer the patient an assessment interview in the department for work integration. During the interview, workers were informed about ways to reduce their workload and, if necessary, solutions were evaluated together with the employer. In addition, workers received assistance from the work specialists in applying to DI for an increase in disability pension, to cover the health-related loss in income.

*Nr.20 Physician*

*I think our annual check-ups are moments when we look together: “Is everything still okay?”*

*When patients are 55 years old, this is a phase in which they often say: “I just can't do it anymore”. Then you have to check with the employer and the cost bearer whether the current workload is still reasonable or whether it needs to be reduced. Then we may involve the work integration department of the specialized SCI rehabilitation center*.

Persons with ABI on the other hand showed a decrease in cognitive performance or emotional control, and increased fatigability. “Red flags” indicating an increasing overload at work manifested as an increasing number of sick days, frequency of errors, withdrawal from social contact with colleagues, or decreased emotional stress tolerance. They usually consulted their family physician, who prescribed rehabilitation in case of need.

Changes in the workplace were the most frequently expressed issue that influences sustainable work for persons with ABI. Warning signs are primarily changes in supervisors or employers. The new managers are unaware of, or unwilling to continue with previous agreements on work adjustments for the worker. The second critical area of change involved reorganizations, organizational adjustments or the introduction of new technologies, with the consequent risk of overwork and the worker feeling left behind, as described in the following quote:

*Nr.10 Vocational specialist*

*There may also be changes in the workplace that the worker is not on board with. And what we unfortunately find time and again is that changes in supervisors are not always good. Unfortunately, we have repeatedly found that this new supervisor then said, no, I don't really want to work with this person anymore*.

*Nr.7 Social counselor, NGO*

*I have a very concrete example in mind right now: A chef who was the head chef of a large canteen with 200 persons and after the brain injury he was reintegrated as a normal chef, slowly, slowly, in a retirement home in the city. We spoke to both the head chef and the director of the home for the older adults. The two switched after three to four years and then suddenly a phone call came from Mr. T.: “They don't understand me anymore, they think I don't work enough, I have to do something else”. His tasks were already defined somewhere in the files, but the newcomers didn't even look at them*.

For persons with SCI, workplace changes were only mentioned in connection with loss of wheelchair accessibility or parking for persons with disability.

#### Search for help – who and where

Many good services exist for initial integration, but support options for work problems that arise after complete integration are less clearly defined. In working-life after the completion of the integration, the vocational integration specialists and the insurance specialists are no longer present. Problems are identified by the worker or the employer, or in rare cases for persons with SCI, during the annual medical checks.

*Nr.13 Administrator, disability insurance*

*So, if the integration is successful, then we conclude the professional measures on the part of vocational counselor. And there is no long-term control, in that sense*.

*Nr.11 Case manager, disability insurance*

*What does the DA know about the work situation of their clients after the integration? It is actually a topic that is discussed again and again, also at national level, that the DA actually, unfortunately, knows far too little about the sustainability of professional measures. Because, yes, that has something to do with the fact that it is relatively time-consuming to follow up on the insured*.

When asked who the worker or the employer could turn to when problems arise, patient organizations were the most frequently mentioned by participants. For persons with SCI, the work integration department of the specialized rehabilitation center was explicitly mentioned. While the support provided by the SCI patient organization was perceived by those concerned to be comprehensive and to meet most of the needs of the workers in need and their employers, Fragile Suisse - the patient organization for persons with brain injury and their families- was perceived to be helpful but is understaffed and underfunded given the number of persons seeking help.

Most experts advised involving the DI as soon as possible, especially in the case of increasing health problems. In this case, they also pointed out that DI representatives base their decision on current health certificates. Therefore specialized health professionals, such as neurologists, neuropsychologists, or paraplegiology specialist physicians should be contacted to certify the health problems and guide the next steps.

Surprisingly, knowledge of services offered by employer organizations or by local support networks for employers and workers with disabilities, or by private job coaches was scarce.

#### Contracting and financing of support

A major topic for the experts was the financing of vocational measures after the completion of work integration, in the event that new problems should arise.

While medical and medical-rehabilitative measures, as in the case of infections or decubiti in persons with SCI, or headaches and mental disorders in persons with ABI are still covered by health insurance or accident insurance, the financing of measures in the workplace varies. For example, if a person's ability to work is less than 8 h per week, DI funding for work integration measures such as job coaching, training or job search assistance, is limited. Another problem is the financing of vocational support (by the DA) after the integration phase has ended. According to the law, DA is only responsible if the problems that occur are due to a further, permanent and officially diagnosed decrease in health. Problems that are due to change at the workplace are not supported. Nevertheless, diverse support programs and funding sources were named in case of need from professionals:

*Nr.3 Consultant*

*Our big plus with mentoring is that we do not have referring agencies. We are not financed by DI. That means we are very free, we are very unbureaucratic. We can set the rhythm ourselves together with the person concerned or the employer. That is the absolute gem for me. My biggest challenge now is a long waiting list in mentoring and refinancing. Exactly, the freedom means for us to finance our support constantly by fundraising*.

*Nr.8 Job coach*

*At present, I provide long-term aftercare for one person, whereby the employer privately pays for my support because it is simply worth it to him. In theory, he wouldn't have to.*

*I am convinced that follow-up support should be financed by the DI because it is simply sustainable in the longer term*.

*Nr.7 Social counselor, NGO*

*Coaching in the workplace? There are several neuropsychologists who can provide such support. There's always a bit of a problem with financing in the long-term phase. Workplace coaching is often not a mandatory service, and is mostly not recognized after the end of the work integration phase*.

### Theme 3: knowledge, experience and attitude of the professionals

The knowledge, experience and attitude of professionals was discussed by most participants – ABI and SCI specialists alike – as central to a successful cooperation towards sustained employment. This statement applies for the integration phase and for long-term employment.

All professional groups emphasized the importance of knowledge in the field of work integration, as well as knowledge about the long-term consequences of a brain or spinal cord injury in daily life. While good knowledge is essential for health and work professionals to provide the best possible support to the client and his or her environment and to coordinate processes smoothly, increasing experience enabled them to use growing professional networks or to find creative solutions, e.g., for individual adaptations at the workplace.

For DI professionals or insurance company case managers, a good knowledge of the disease was seen as a prerequisite to organize administrative processes in a need-based and efficient way and to plan financing strategies, according to the chronic nature of SCI or ABI in the long term.

Most professionals agreed that an open, appreciative and trusting attitude on the part of all professionals is a basic prerequisite for successful cooperation and coordinated support of the worker on the path to professional integration and sustainable work. Lack of experience and specific knowledge about the daily reality of people with a chronic illness, or about administrative or legal procedures affecting them, on the other hand, can lead to an overestimation of the employee's capacity. They are not taken seriously or their needs are ignored. Furthermore, study participants have emphasized that the technical overload of professionals, just like a chronic lack of time, leads to wrong decisions and sometimes long histories of suffering on the part of employees.

*Nr.7 Social counselor, NGO*

*It is often the family doctor with whom the patient is still in contact after the end of professional integration, which is the same as with the psychiatrist. Unfortunately, there is only a small group that has experience with the consequences of brain injuries. This is understandable, because they have to deal with many other things. However, that's one reason why they often overlook emerging issues or don't have an appropriate strategy to offer. Additionally they are also at odds with the new tariff structures, which say you can only speak to the patient for 20 min. Then you can forget about it. There just isn't enough time to identify and address work problems*.

*Nr.1 Vocational specialist, OT*

*Especially when it comes to persons with brain injuries, I often have the feeling that DI workers are also overwhelmed. They reach their limits with these persons. They don't know enough about the symptoms and often don't realize that persons with brain injuries still have serious problems that show up in subtle ways in everyday life*.

Advisors from patient organizations in particular complained that some specialists lacked awareness and empathy for the long-term life situation of their clients during vocational rehabilitation and work integration. The attitude of these professionals stems from the fact that they mainly identify with the goals, financial and time requirements of their legal clients (e.g. DA or other payers). Coupled with little professional experience with persons with ABI or SCI, they take a performance-centric approach to integrate their clients into the workforce as quickly as possible, while putting aside the sustainability-focused, person-centric approach, demanded by most professionals in the interviews.

This short-term integration policy is welcomed by many cost bearers due to the political pressure to save in the health and social services, especially since in Switzerland no success evaluations for the sustainability of professional measures are currently carried out by the cost bearers.

*Nr.4 Consultant, Fragile Suisse*

*I think there are many providers of professional measures who have no idea. Some, for example, work indirectly for DI under pressure to get workers back to work as soon as possible. You can do that, you can say: “We are job coaches, we accept orders from the DI, for which we receive a bonus if the worker works again after three months”. But these persons don't care if the worker can still work after a year*.

## Discussion

In this study we identified and explored factors that enable or hinder sustainable employment of persons with ABI or SCI that are considered relevant for professionals contributing directly to the workers sustainable employment. Participants identified three main themes: (1) a good start predicts a successful working life, a theme that addresses the value and impact of initial work integration for sustained employment in the long-term and its critical influencing factors, (2) change is a challenge to foster sustainable long-term employment, and (3) the knowledge, experience, and attitude of professionals. In theme 3, the participants addressed the importance of a good qualification and an open, person-centered attitude of professionals who provide services to the persons concerned.

Surprisingly, the same themes and influencing factors were found to be relevant in both groups, ABI and SCI. Nevertheless, differences were found in the measures prioritized or perceived as important by participants. (Supplementary material 2). These differences were mainly due to different physical, mental and psychological effects of the respective injury and its functional consequences, with persons with ABI experiencing mainly neuropsychological problems and fatigue, and persons with SCI mainly experiencing secondary health problems such as pain, pressure ulcers, and infections.

Differences in the organization of work integration of the two diagnostic groups may be partially explained by differences in care service delivery processes. Most persons with SCI experienced initial rehabilitation in one specialized rehabilitation center, with early contact through the vocational integration department, followed by a seamless transition to a highly professional, well-coordinated vocational integration. This integrated service pathway from initial rehabilitation to work integration and beyond, provided by the specialized services of the Swiss paraplegic foundation and their specialized Rehabilitation center, may be one reason for the high work participation of persons with SCI in Switzerland compared to other countries (14). Rehabilitation for persons with ABI, on the other hand, is provided in many different centers throughout Switzerland. For these persons, the transition from initial rehabilitation to a more advanced vocational rehabilitation or work integration measure depends on the services offered by the respective rehabilitation clinic or the availability of more advanced vocational measures in the region. As only undetailed, estimated figures on the frequency of ABI exist in Switzerland (13), it is unclear how many persons with minimal to moderate brain injuries and with mainly neuropsychological or mental health problems, return to work without knowing that vocational intervention measures exist for them. If they fail at work, they often have a long way ahead before the consequences of the brain injury are recognized and DI only then is informed and may support vocational integration measures.

In the process of work integration however, as well as in the case of an intervention in the long-term phase, case managers and work integration specialists favored a person-centered approach, irrespective of the diagnosis. Professionals favored the Supported Employment model (SE) or process-oriented guidance approach, where SE can be described as “a model based on the belief that anyone seeking paid employment can achieve it with the right support”. SE is a successful, accepted, and flexible tool for assisting persons with a disability find meaningful and reasonably compensated work.” (33). Supported Employment was favored by professionals independent of the health condition of the person and is consistent with international recommendations (35–37).

Despite a high level of approval for coordinated professional integration under the leadership of a job coach or case manager, there are also professionals, namely physicians, who feel limited in their professional competence. On the other hand, they often have little time and limited knowledge to take care of coordination tasks in vocational integration. Recognized further training courses on the topic of vocational integration, with a focus on role understanding and the division of tasks specifically for general practitioners, could help doctors to be more effectively involved in the rehabilitation team. Such training could also help physicians to identify risk factors in the workplace in the long-term at an early stage, and to refer patients to the right services promptly if necessary.

In our study, we asked participants about the factors that enable or hinder sustainable employment for persons with ABI or SCI. Nevertheless, most of the statements referred to the phase of work integration. This reflects in particular the work reality of nearly half of the employment specialists, job coaches, and insurance specialists, which often ends when the integration phase is completed. Medical professionals and persons and services of patient organizations or NGO's, on the other hand, also reported on their experiences with workers from the long-term phase, but had little knowledge on early signs indicating work-endangering situations. Our study participants' knowledge was limited about resources that can provide guidance and support in case of problems at work.

In contrast to the professionals themselves, individuals with ABI or SCI or their employers mentioned professionals' lack of knowledge about the consequences of ABI or SCI, incorrect advice on legal issues, a lack of time resources, and sometimes negative attitudes toward disabled workers among professionals, but did not focus on these issues (36, 37). This could be because employees and employers experience difficulties with professionals mainly during the post-injury and work integration phases. In the phase of long-term sustainable work, however, professionals are hardly present for affected employees and employers and therefore play only a minor role in the discussion of risk factors for long-term job retention.

When comparing the findings from this study in the Swiss context, factors reported as crucial for successful work integration and sustained employment such as early onset of vocational measures (38), importance of the personal characteristics, self-management and self-advocacy (39–41), the importance of a well-coordinated interdisciplinary vocational rehabilitation (42), and the importance of a supportive employer and colleagues were also reported in international studies (43). The same is true with Supported Employment as one of the most popular approaches (37, 44, 45).

Financing of work integration measures in general might be an issue in several countries, but the specific issues discussed from participants in this study might refer explicitly to the Swiss disability system (46). The same might apply to long-term work interventions such as receiving partial disability pensions and the possibility to adapt them in case of health-related increased or decreased earning ability, as they are unique features of the Swiss social and disability policy.

However, an easily accessible, lifelong support system for persons with chronic health conditions might be a promising approach to consider internationally to effectively address work-threatening factors for persons with ABI or SCI, such as a deterioration in health, or in the event of organizational or personnel change in the company (47–49).

### Strengths and limitations

One main strength of our study is that we collected data from a wide range of professionals representing the stakeholders directly involved in enabling sustainable work for persons with ABI or SCI. This speaks to the diversity of input that we gathered in this study. Another strength is the inclusion of professionals working with two different health conditions, ABI and SCI, which together cover the vast majority of movement-related, cognitive, psychological, and social functioning-related problems typically associated with neurological disorders.

However, our study has also some limitations; there is a risk of bias due to the convenient recruitment strategy and purposive sampling. Although participants met the inclusion criteria of having insight into sustained employment in the long-term of persons with ABI or SCI, during the interviews, integration specialists, including case managers in big companies showed limited insight beyond initial integration. Despite an extended search, no work professionals could be found who look after clients with work problems, mainly in the long-term phase. Employees of the human resources department were surveyed as part of the “employer perspective study,” since in Switzerland they primarily represent the employer's perspective to employees. On the other hand, this fact points to the need to empower and educate employers and the workers to identify problems early, as after the end of the integration phase, they are on their own. Another factor that may have influenced the results differently in the two groups is the demographic characteristics of the persons with ABI or SCI, such as age, age at injury, gender, and education. However, because there are no official figures on persons with ABI in Switzerland, we cannot analyze these differences further.

### Practical, policy and research implications

This study identified factors common to ABI and SCI, such as a need for professional support for health-related work problems in the long-term. Such factors may point to a generic gap in service provision and may also be relevant to other health groups experiencing similar problems in functioning. Identified gaps may further help to improve services, or be addressed in the legislation on financing work disability-related measures. Identified differences in the organization of professional integration services, such as integrated care programs for persons with SCI in specialized rehabilitation centers and, in contrast, often fragmented care for persons with ABI, may inform new care strategies for persons with a disability. Findings of this study strongly support a person-centered approach for work integration measures to reach sustained employment that is ideally based on the Supported Employment model. Job coaches, case managers, and social workers in the field of work should demonstrate that they keep their skills up-to-date through lifelong learning (50). In addition, providers of vocational integration measures and, in particular, insurance agents responsible for persons with SCI or ABI should have a sound knowledge of the consequences of these health disorders. This is necessary so that they can assess the benefits of integration measures in individual cases and provide timely support. Ideally, professionals or teams with specific expertise should be assigned to care for individuals with ABI or SCI. Specialist training is currently offered in Switzerland by universities, private providers and by patient organizations. However, in the case of DI or private insurances, diagnosis-specific care could mean that persons with a rare injury, such as SCI, would be cared for in regional or even cantonal teams.

Participants also pointed out that ABI and SCI must be seen as chronic conditions with a lifelong impact on functioning that can change over the years, what is strongly supported by international evidence (47, 48). A legal basis for easily accessible support outside of pure medical treatment, including employment measures, should be further investigated. This offer should also take into account the positive social and health effects of participation in the labor market, and not only the wage to be earned; work integration measures should consequently also be considered for persons with severely limited earning capacity and full disability pension (49, 51). The integrated life-long support offered by the Swiss paraplegic foundation for persons with SCI, could provide a role model for other health conditions including ABI. The greatest challenge for the implementation of such an offer is secure financing throughout the work-life. Long-term funding would require a change in the law and also a major shift in administrative processes away from event-based case management and funding to a lifetime model.

As professionals have limited knowledge about signs indicating problems at work in the long-term phase early on, directly affected employees and their employers including human resource representatives as experts on sustainable work should be included in further research. A participatory action research (PAR) approach that includes stakeholder dialogue lends itself to enabling providers, payers, and policymakers, together with those directly affected, to contribute their insights to the development of constructive solutions (52). Longitudinal studies addressing the long-term phase and studies investigating the biographies and CVs of workers are also important as a next step to better understand the development of problems. Implementation research can further play an important role in translating findings and recommendations into the organization of service delivery or long-term support for people with ABI or SCI. Additional research focusing on the sustainability of work intervention measures, and the need for support in the long-term in Switzerland may help work integration services and funding agencies to improve their services.

## Conclusion

From the perspective of professionals, most of the support factors for sustained employment of persons with ABI or SCI, relate to the work environment and the personal characteristics of the person. They are fundamentally very similar. The differences arise mainly from the physical, mental, and psychological effects of the particular injury and its functional consequences.

Both, knowledge of “red flags”, signs that warn of work-hazardous situations, and knowledge of services and persons, who can provide long-term counseling or support for workplace problems, is person-dependent and often limited.

Raising awareness and empowering individuals and their employers about problematic changes in the future is therefore critical for sustainable employment.

## Data Availability

The datasets presented in this article are not readily available because the Data (interview transcripts) contains information that may lead to an identification of the participant. Full anonymization can not be guaranteed without deleting essential parts of information. Requests to access the datasets should be directed to monika.finger@paraplegie.ch.
