# Supplementary material for: Factors influencing sustainable employment of persons with acquired brain injury (ABI) or spinal cord injury (SCI): A qualitative study evaluating the perspective of health and work professionals
Source: Front Rehabil Sci. 2023 Jan 20;3:906567. doi: 10.3389/fresc.2022.906567 (PMC9895944; doi:10.3389/fresc.2022.906567)
Supplement: Supplementary file 1 [file Datasheet1.zip › Appendix 1.DOCX]

**Guidelines for interviews with specialists/work, health, payers, social insurance**

**Preamble**

An individual interview is conducted with professionals at their workplace or at a jointly chosen location.

Professionals are health or work professionals who have cared for, trained or coached people with a spinal cord injury or a brain injury during their professional integration and possibly later at their regular workplace. The work of the professional may also include a mediation function between the employer/workplace and the coachee.

The interview serves to record the experiences of the participants and thus to gain knowledge on the subject of "long-term, sustained, satisfied work with a spinal cord injury or a brain injury in Switzerland".

In a guide, questions were selected and arranged in advance so that the course of the conversation is stimulated but structured at the same time.

The interview, including the introduction to the topic, is conducted by a trained interviewer. The conversation is digitally recorded. The interviewer also notes key points of the conversation.

These notes help the researchers in structuring the interview and later in the exact transcription of the electronically recorded conversation, as well as in the subsequent evaluation.

It should be ensured that the participants are anonymously marked in the notes and the subsequent transcript (with age and gender).

**Objecti**v

- to identify and explore the factors that enable or hinder sustainable employment for persons with ABI or SCI in Switzerland from the perspective of professionals.

(i) to explore factors and underlying mechanisms that promote or hinder persons with SCI or ABI to stay sustainably at work after their injury, and

(ii) to identify persons or services that may provide guidance or assistance for the injured workers if necessary during their work life.ve:

|  | Theme | Material | Time |
| --- | --- | --- | --- |
|  | Material |  |  |
|  | - Audio recorder - Conversation Log / Notepad - Topic pie chart template - Letter of acceptance |  |  |
|  | Introduction |  |  |
| 1 | - Welcome and thank you for your willingness to participate - Introduction (SPF / project team member of the study, aim of the study, conduct of the interviews (topic, duration) - Notices of privacy and anonymity, recording, - (If required) Sign the consent form - Interest in your point of view, open and spontaneous answers, there is no right or wrong | Informed consent form | 5‘ |
|  | Positive experiences / challenges |  |  |
| 2 | 1. Introduce yourself: job, position in the company, how long there, work experience? 2. What is your experience with clients with spinal cord injury or brain injury? 3. What do you think it takes for people with paraplegia or a brain injury to be able to work successfully in the long term?   *Let the participant express his thoughts and experiences. Once he/she finishes, present the topic circle diagram to further stimulate associations.*  *Topic circle diagram based on SR: Are there still topics in these areas that are important for positive cooperation? E.g. person himself, work performance, workplace-related, environment, social system*   1. After you have received the additional information: In your opinion, are there additional success factors that lead to an employee with spinal cord injury or a brain injury being able to be integrated into a workplace in the long term? | Topic circle diagram | 15‘ |
|  | Challenges |  |  |
| 3 | 1. What are the biggest challenges for you as a professional?   *Present topic pie chart:* Do you see unaddressed challenges in these areas?   1. What is/were the biggest challenges for you in working with people with spinal cord injury or a brain injury in terms of sustainable employment? 2. What are "red flags" or warning signals for an imminent long-term absence from work by the employee or even for a permanent exit from the job market? | *Topic circle diagram* | 15‘ |
|  | Need for support / gap |  |  |
| 4 | 1. What (what measures) can you offer to support the person concerned or the employer to stay sustainably at work? 2. What other support options do you know / use?   *Deepening: support by whom, how, type of support, expectation of the result of the support?*   1. In your view, what other offers/measures/legal foundations would be needed? 2. What does it take for a long-term win-win situation for employers and employees? |  | 10‘ |
|  | Abschluss Interview |  | 45‘ |
| 5 | 1. From your point of view, are there any additions or recommendations that you would like to give us / something that we must not forget? 2. Thanks and farewell |  | 2‘ |

**Topic diagram**

**
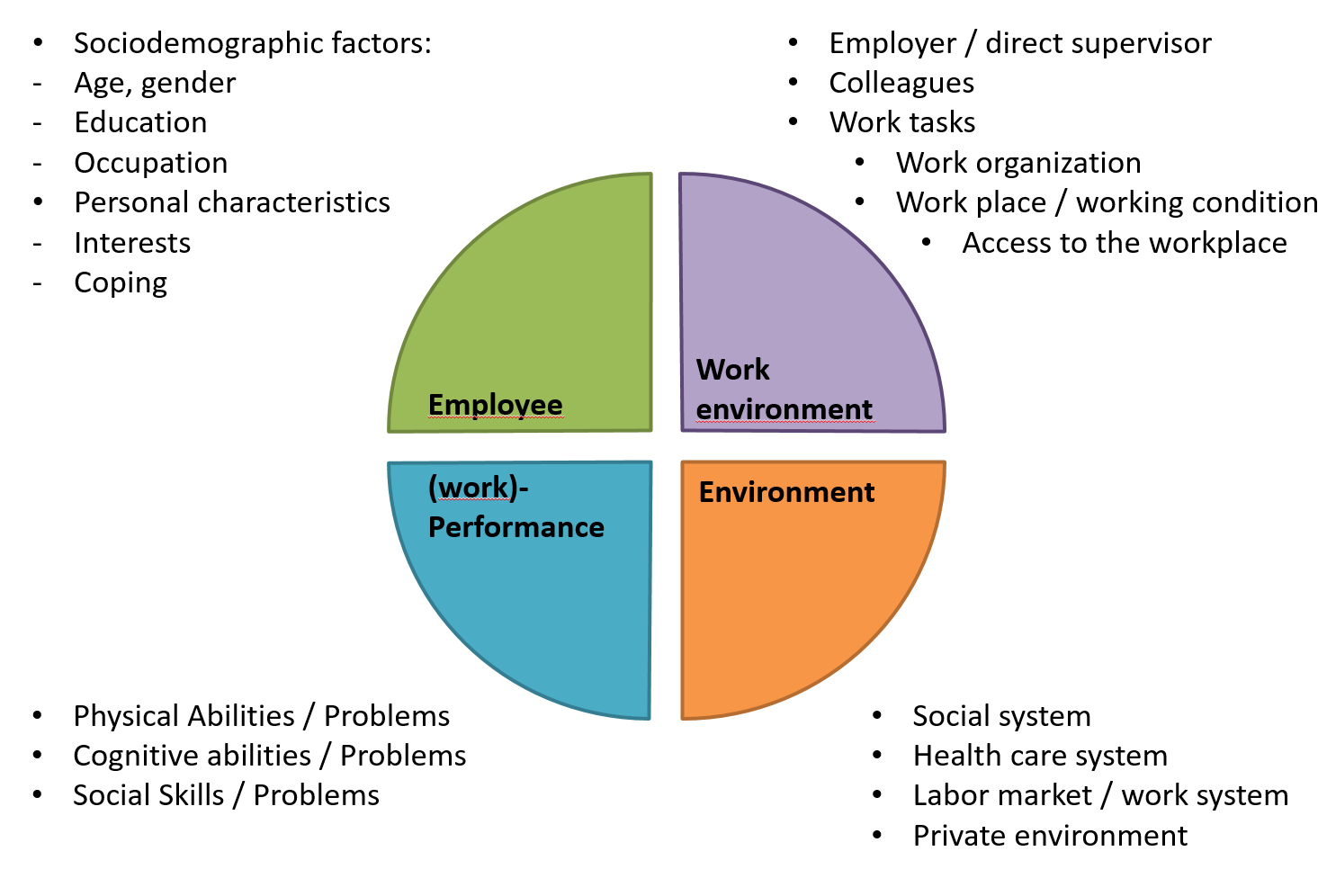
**
