# Supplementary material for: Factors influencing sustainable employment of persons with acquired brain injury (ABI) or spinal cord injury (SCI): A qualitative study evaluating the perspective of health and work professionals
Source: Front Rehabil Sci. 2023 Jan 20;3:906567. doi: 10.3389/fresc.2022.906567 (PMC9895944; doi:10.3389/fresc.2022.906567)
Supplement: Supplementary file 1 [file Datasheet1.zip › Appendix 2.DOCX]

**Supplementary Table 1: Factors that support and hinder sustainable employment.**

| **Main Theme / Subthemes** | **Factors included in the theme** (+) supporting factors; (-) hindering factors | **Exemplary citation** |
| --- | --- | --- |
| Theme 1  A good start predicts a successful working life | + coordinated vocational rehabilitation (ABI&SCI)  + patient centered, individualized approach (ABI&SCI) | Many factors and actors play a role in ensuring that a worker with an SCI can be sustainably working at the end. The worker himself, the disability insurance, the accident insurance, the whole team of doctors, health professionals and job coaches, also the employer and the family environment. It is crucial that those involved are in regular contact and inform each other, and not that everyone tends their own garden. |
| 1.1 Time is crucial | + early onset of vocational interventions (ABI&SCI)  + learning to cope with life with a (ABI&SCI)  + strong motivation to work (ABI&SCI)  + ability to self-reflect on strengths and weaknesses (ABI&SCI)  + time to get (physically and mentally) ready for work (ABI&SCI)  + engaged employers (ABI&SCI)  + support of retraining in the new profession (SCI)  + establish a viable work-life balance (ABI&SCI)  +/- financing of vocational measures until end of vocational integration (up to several years) (ABI&SCI)  - delayed vocational rehabilitation (ABI) | Any person with a brain injury must first learn to know what works and what doesn't. It is also very important to have the ability to recognize your limits.  As an example; Sometimes I use a pizza analogy with my patients; I explain: “Ok, this piece of the pizza indicates the amount of time you can sleep each night and you shouldn’t sleep any less than this amount. You also need to reserve another piece (time) for regular bodily functions and self-care in the morning.” And the only piece left of the pizza indicates the amount of time you have to somehow distribute between eating, working, social and personal activities. |
| 1.2 Coordination of all parties involved | + good coordination across settings (ABI&SCI)  + integrated care from first day of rehabilitation until work integration (ABI&SCI)  + coordinated interdisciplinary, inter-stakeholder team which has defined roles and person in charge (case manager, job counselor) (ABI&SCI)  - time needed for meetings (SCI)  - professionals feel restricted in their (ABI&SCI) | Who is the leader? There are a whole range of players, each with individual roles. Basically, in the case of an accident, we would contact? the accident insurance. However we more commonly turn to the work integration specialists at the rehabilitation center, who’s role is to establish and maintain contact with the employers.  The real problem is that we as doctors no longer have as much say in the matter. As an example; health insurance companies have the right to refuse a sick note. I can issue a sickness certificate for the patient, and then some administrator from the insurance company can say, “No, that doesn't apply”. |
| 1.3 Communication and knowledge transfer across stakeholders | + direct exchange of information across stakeholders (ABI&SCI)  + information and education of employer and work colleagues, about consequences and nature of health condition. (ABI&SCI)  + Professional as mediator between affected person, work place and insurances. (ABI&SCI) | It is vital that the environment and surroundings must understand exactly what it means to have a brain injury. [..] And if it is at all possible that the injured worker can adapt to the requirements of the workplace. I think the employer and colleagues need to be educated on what the proper expectations are from someone with a brain injury, they must have a positive attitude and understanding to best support their employee/peer.  Often, I think the affected person would benefit the most by having a companion who can be an advocate for them or if the affected person is capable, then they can personally express their own needs.  I don't want to intimidate people, I want to educate and promote understanding by pointing out: "Hey, this is a guy who you can expect a good performance from, but there are some specifics that you should be aware of due to his injury." I actually start every coaching assignment with a talk at the workplace by saying: "I'm going to inform you about paraplegia for an hour.” |
| Theme 2: Change is a challenge – for better or worse | + career development (SCI)  - deterioration in health (ABI&SCI) | What I've sometimes experienced is that people came to me for counseling after years of working and say: I'm 40 years old, I've been back at work for 8 years, things are going well, but I can't advance anymore. I'm blocked, my career is over for me, I can't develop any further". This is only partially true. It is important to look at what other developmental opportunities are still available. [...] Because standing still is not burnout, it's like a bore out. |
| 2.1 Threats to sustainable employment- and red flags | + annual health checks (SCI)  + adaptation of work conditions (ABI&SCI)  - decrease in physical abilities (SCI)  -decrease in cognitive performance, emotional control (ABI)  - secondary health conditions (SCI)  - increased number of sick days (ABI&SCI)  - changes in supervisor or employer (ABI)  - forgotten knowledge of consequences of health at the work place (ABI&SCI)  - reorganizations at the workplace (ABI)  - introducing new technologies (ABI)  - loss of wheel chair accessibility or parking space at the workplace (SCI) | Yes, patients often report physical complaints. As an example, If I hear 'Aha, they are reducing their therapies, or they are reporting an increase in urinary tract infections or pressure sores, then we in the outpatient clinic know that we have to ask "What's going on?" because such an increase in health complications often indicates that they are not investing enough time in their health situation.  … of course, it is possible that they experience bladder changes, dysregulation and blood pressure crises repeatedly or something similar. Times have changed and self-care in the morning tends to take much more time than it used to. This means the person gets to work much later. If the work output or the working time is then reduced, this is usually not as bad, but only if the quality of work is still acceptable. Consistency is also very important for employers as it is not okay for a worker to do a fantastic job one day and then do a poor job the next, or if they don’t even show up for work and don’t even call in sick.  When I notice that patients stop working due to health problems, then the situation becomes critical. This is not good for self-confidence, coping, and not good for the relationship with the employer and colleagues. In the beginning the employer and colleagues may understand, but after the fourth pressure sore or sixth urinary tract infection the affected worker will take even longer to return to work and the remaining colleagues will have to pick up the slack. In addition, the patient in this situation often loses the support of the employer. |
| 2.2 Search for help – who and where | + patient organizations (ABI&SCI)  + health care specialists: e.g. physician, psychiatrist, (neuro)psychologist (ABI&SCI)  +/- disability insurance and other insurances (ABI&SCI)  - professionals who support work integration are no longer responsible or available (A)  - knowledge on local support networks and Non-Governmental Organizations (NGO) is scarce (ABI&SCI) | [Do you know supporting institutions or actors?] Institutions not, but actors. An example of actors would be members of the wheelchair club. Whereby, these are often colleagues, or the life counseling service of the Paraplegic Association. The important persons or services are the ones that you meet somewhere and they chat with you. They may also have a professional sense of what you are going through and what developmental stage you are at currently. These people act as sensors that can say: "You know what, I think I would advise you to see a psychologist". |
| 2.3 Contracting and financing of support | + support of patient organizations and NGOs (A&S)  - limited time and financial resources, especially in the patient organizations, NGO's and DI (**A**&S)  - no DA support for reintegration if work ability is less than 50%. (A&S) | Where can an employer go for help if problems arise?  An employer can seek help at the known agencies, such as the DA, ProInfirmis (NGO) or Fragile Suisse (patient organization). Due to the fact that the cases were once with the DA, the DA would be the easiest working partner, because they can simply pull out the dossier. Or the accident insurance, if it was an accident. |
| Theme 3: Knowledge, experience and attitude of the professionals | + knowledge of legal context and health condition of professionals (A&S)  + appreciative, trusting attitude of professionals (A&S) | From my perspective an insurance case manager must have many years of experience to be able to properly perform their expected duties. They must know how to use financing measures to move those directly involved, from the care provider and the care giver to the employer. Where do you invest money to achieve added value for everyone? It requires experience in accident insurance law, as well as disability and health insurance. They must know the liability insurance situation and the applicable law in Switzerland. Our team here consists of four people, and we have the professional experience of 95 years. |
